# A Mechanism for Nerve Cell Excitation by Norepinephrine via Alpha-1 Adrenoceptors: Inhibition of Potassium M-Current

**DOI:** 10.1007/s10571-012-9870-y

**Published:** 2012-08-08

**Authors:** Alexander K. Filippov, David A. Brown

**Affiliations:** Department of Neuroscience, Physiology and Pharmacology, University College London, London, WC1E 6BT UK

**Keywords:** α1 Adrenoceptors, Norepinephrine, Phenylephrine, M-current, Potassium current, Sympathetic neurons

## Abstract

Some of the excitatory effects of norepinephrine on central neurons are mediated by alpha-1 (α1) adrenoceptors. These receptors are coupled to the Gq family of G proteins, and hence stimulate hydrolysis of the membrane phospholipid phosphatidylinositol-4,5-bisphosphate. Other receptors of this type can excite neurons by inhibiting the subthreshold voltage-gated potassium M-current. We tested this possibility using rat sympathetic neurons transformed to express α1a receptors. The α1 agonist phenylephrine strongly inhibited the M-current recorded under voltage-clamp by 72 ± 11 % (*n* = 4) and in an unclamped neuron dramatically increased the number of action potentials produced by a 2 s depolarizing current step from 2 to 40, without effect on control neurons devoid of α1 receptors. We suggest that this might be a potential cause of the increased excitability produced by norepinephrine in some central neurons.

## Introduction

Alpha-1 (α1) adrenoceptors are widely distributed in the central nervous system (Jones et al. 1985; Day et al. 1997). There they mediate some of the effects of norepinephrine, including a membrane depolarization and enhanced action potential firing (e.g., supra-optic nucleus: Ogata and Matsuo 1986; cultured spinal neurons: Legendre et al. 1988; cerebral cortex and thalamus: McCormick et al. 1991; lateral geniculate nucleus: McCormick 1992).

These receptors are G protein-coupled receptors (GPCRs) that link primarily to the Gq family of G proteins and thereby stimulate hydrolysis of the membrane phospholipid phosphatidylinositol-4,5-bisphosphate (PIP_2_) (Hawrylyshyn et al. 2004). Other receptors of this type can excite neurons by inhibiting the subthreshold voltage-gated K^+^ current termed the “M-current” (Delmas and Brown 2005), which normally acts as a brake on firing activity. However, this action does not appear to have been hitherto detected in mammalian central neurons following α1-stimulation. This may be because most of the cells studied so far did not possess prominent M-currents, or perhaps because the current is not very responsive to α1 receptor stimulation (though tests on peripheral parasympathetic neurons suggest otherwise: Shibata and Taketani 2001).

We have recently had an opportunity to assess the response of M-currents to specific α1-adrenoceptor stimulation using neurons from the rat superior cervical sympathetic ganglion. These have large M-currents (Constanti and Brown 1981) but do not seem to possess an appreciable number of endogenous α1 receptors (Grayson et al. 1998; Dawson et al. 2010). Hence we inserted α1a adrenoceptors by intranuclear cDNA injection, as used for other receptors (see Ikeda 1997; Filippov et al. 2000). We show that this receptor is indeed well capable of inhibiting the M-current, and can thereby strikingly enhance excitability.

## Materials and Methods

Superior cervical sympathetic ganglia were isolated from 18-day-old rats (killed by UK Home Office approved methods), neurons dissociated, plated and cultured in vitro for 1–2 days as described by Filippov et al. (1998). To express α1a receptors, a cDNA plasmid encoding the receptor (provided by Dr. John Pediani, Glasgow University; see Pediani et al. 2000) was injected into individual cell nuclei 4 h after initial plating, together with a cDNA plasmid encoding the jellyfish enhanced green fluorescent protein (eGFP) to mark the injected neurons. For electrophysiological recording, cells were patched with electrodes containing (in mM) 90 potassium acetate, 20 KCl, 3 MgCl_2_, 40 HEPES, and 0.1 BAPTA (pH-adjusted to 7.4 by KOH), and back-filled with the same solution containing 0.125 mg/ml amphotericin B as a membrane permeabilizing agent, to provide “perforated-patch” recording. (This is essential to retain the normal cytosolic constituents and maintain normal M-currents and excitability.) Cells were maintained in flowing Krebs’ solution at 20–22 °C containing (in mM) 120 NaCl, 3 KCl, 1.5 MgCl_2_, 2.5 CaCl_2_, 10 HEPES, and 11.1 glucose (pH-adjusted to 7.3 with NaOH). Recordings were made, data collected and analyzed as described by Filippov et al. (1998).

## Results

### M-Current

**Fig. 1:**
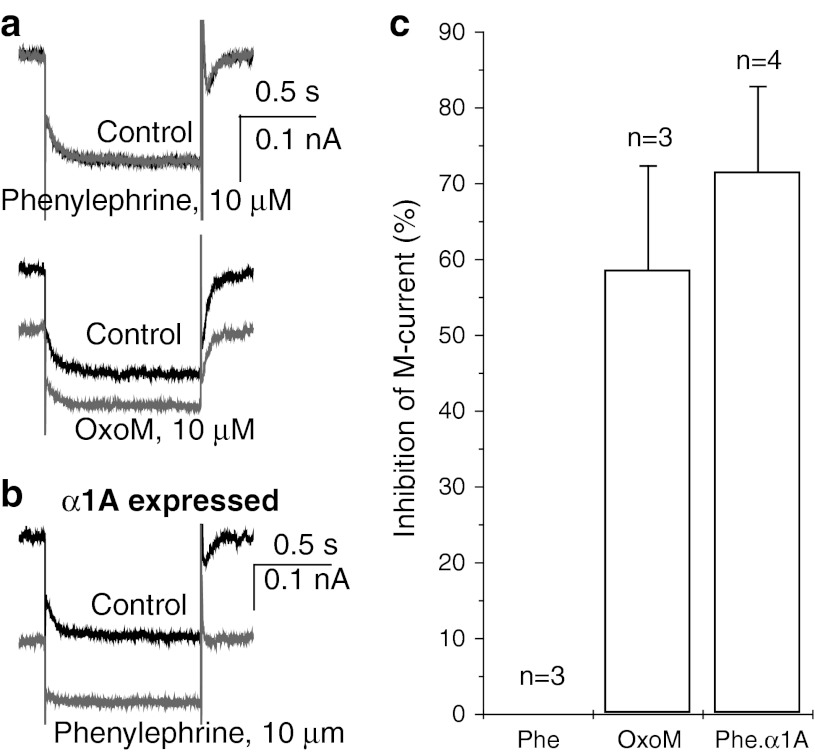
M-current inhibition by oxotremorine-M (oxo-M, 10 μM) and phenylephrine (phe, 10 μM). *Left side* voltage-clamp current records from **a** a normal (wild-type) neuron and **b** a neuron pre-injected with cDNA encoding the α1a-adrenoceptor. Neurons were held at −20 mV to activate the M-current as a steady component of outward current. Records show responses to a −20 mV 1 s hyperpolarizing step. M-current deactivation is seen as a slow inward current-tail. **c** *Bar charts* showing mean% inhibition measured from deactivation current-tail amplitudes. *Bars* SEMs, *n* number of cells tested. Currents reverted to control on drug washout. Note that phenylephrine inhibited M-current in α1a-expressing neurons but not in control neurons

When these sympathetic neurons are clamped at a depolarized membrane potential, potassium M-channels open and generate a steady outward current (they do not inactive). Then when the cells are briefly hyperpolarized M-channels slowly close, producing a slow inward tail-current (see Fig. 1a). The amount of M-current lost during the hyperpolarization can be measured from the initial amplitude of this deactivation tail-current at the point of hyperpolarization. This procedure isolates M-current from other membrane currents (Adams et al. 1982a).

In neurons that had not been transformed to express α1a adrenoceptors, the α1-agonist phenylephrine (10 μM) had no effect on either the amount of steady outward current or the amplitude of the deactivation tail-current (Fig. 1a, top trace; Fig. 1c). In contrast, in the same neuron the muscarinic acetylcholine-receptor agonist oxotremorine-M (oxo-M, 10 μM) clearly reduced both steady outward current and deactivation current-tail (Fig. 1a, lower trace), signaling M-current inhibition (see Adams et al. 1982b). Mean inhibition in three such neurons measured from the extrapolated initial amplitude of the deactivation tail-current (Adams et al. 1982a) was 59 ± 14 % (Fig. 1c).

In contrast to the negative effect of phenylephrine in Fig. 1a, this α1-agonist clearly did reduce the M-current if a neuron had been pre-injected with α1a receptor cDNA (Fig. 1b), to a mean extent of 72 ± 11 % (*n* = 4; Fig. 1c). Thus, in an α1a-expressing neuron, phenylephrine inhibits the M-current just like a muscarinic agonist.

### Excitability

**Fig. 2:**
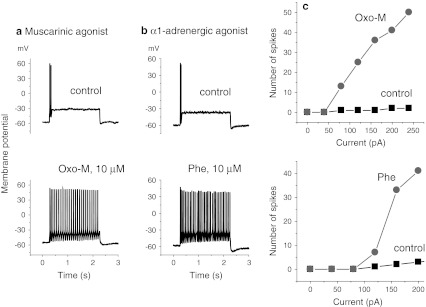
Effects of **a** oxotremorine-M (oxo-M, 10 μM) and **b** phenylephrine (Phe, 10 μM) on action potential firing in two neurons induced by 2 s depolarizing current injections (120 pA in **a**, 160 pA in **b**). Neuron A: wild-type; neuron B: pre-injected with α1a cDNA. **c** Plots of the number of action potentials (“spikes”) recorded in 2 s (ordinates) against the amplitude of the depolarizing current injection (abscissae) for the two cells illustrated in **a** and **b**. Discharges reverted to control after drug washout

M-current confers strong spike-frequency adaptation on these neurons, so one effect of M-current inhibition is to facilitate repetitive firing during sustained depolarization (Brown 1983). Figure 2a shows such an effect of oxotremorine-M. A 2 s depolarizing current injection initially generated only two action potentials at the beginning of the pulse but a sustained train of action potentials after adding oxotremorine-M, rising to 50 action potentials (25 Hz) with increasing current injections (Fig. 2c). Phenylephrine had no effect on induced action potential firing in normal cells but precisely replicated the effect of oxotremorine-M in a neuron pre-injected with the α1a cDNA (Fig. 2b, c). Thus, M-current inhibition by α1a-adrenoceptors would be expected to increase neuronal excitability.

## Discussion

These experiments show that, when present in a neuron, α1-adrenoceptors are well capable of strongly inhibiting the M-current and greatly increasing neuronal excitability, just like an endogenous Gq-coupled GPCR such as the muscarinic M1-receptor.

It could be argued that this is an artifact of receptor overexpression, and that any endogenous α1-receptors are somehow insulated from exerting such an effect. For example, muscarinic receptor overexpression in these neurons can overcome endogenous barriers that otherwise restrict downstream Ca^2+^-signaling pathways (Zaika et al. 2011). However, we think this type of exclusion zone unlikely to confer resistance of M-channels to inhibition, since, in previous tests, we found that overexpression of the small number of endogenous P2Y1 purinoceptors, while amplifying the signals, did not qualitatively alter their actions on M-channels and Ca^2+^-channels (Filippov et al. 2010). Further, endogenous α1-adrenoceptors have been reported to inhibit M-currents in other peripheral neurons (Shibata and Taketani 2001).

In previous experiments on central neurons (see “Introduction”), the depolarization produced by α1-receptor activation was usually accompanied by a reduced K^+^ conductance but in only one case (cultured embryonic spinal neurons: Legendre et al. 1988) did the depolarization show some, though not all, of the properties expected for M-current inhibition. Probably M-currents were insufficiently prominent in the other cells tested. Notwithstanding, bearing in mind the wide distribution of both M-channels and α1 receptors in the brain, it seems likely that more of the α1-mediated effects of norepinephrine on central neurons will prove to be caused by M-channel inhibition when examined in detail. There are only a few thousand noradrenergic neurons in their main site of origin in the locus coeruleus, but their axons ramify widely throughout the c.n.s. (Berridge and Waterhouse 2003), and their activation of α1-receptors are thought to be involved in many aspects of central noradrenergic function, including memory and cognition (Gibbs and Summers 2002; Ramos and Arnsten 2007), sleep (Berridge et al. 2012), depression (Stone et al. 2003), and pain (Pertovaara 2006). Hence, further test for M-current inhibition would seem worthwhile. From the data of Legendre et al. (1988), a plausible start-point might be the spinal cord.
